# A Two-Phase, Joint-Commuting Model for Primary and Secondary Schools Considering Parking Sharing

**DOI:** 10.3390/ijerph19116435

**Published:** 2022-05-25

**Authors:** Huasheng Liu, Yuqi Zhao, Jin Li, Yu Li, Xiaowen Li, Sha Yang

**Affiliations:** College of Transportation, Jilin University, Changchun 130022, China; jtliuhs@jlu.edu.cn (H.L.); yuqiz21@mails.jlu.edu.cn (Y.Z.); yuli20@mails.jlu.edu.cn (Y.L.); lixw1716@mails.jlu.edu.cn (X.L.); yangsha1718@mails.jlu.edu.cn (S.Y.)

**Keywords:** student commute, joint-travel model, route planning, algorithm design, exhaust emissions

## Abstract

In light of the traffic congestion and traffic environment problems around schools that are caused by students commuting by car, this paper explores an efficient and feasible student commuting travel plan. Based on the ideas of “public–private cooperation” and “parking sharing”, combined with the characteristics of the family travel chain during the commuting period, a joint-commuting model of “private car and school bus” is creatively proposed. On the basis of considering the travel cost of parents and the operating cost of school bus, a two-phase commuting travel model for primary and secondary schools is proposed, and an algorithm is designed. The validity of the model is verified by an example and sensitivity analysis. The results show that the total time cost can be reduced by 23.33% when the private-car commuting mode is converted to the joint-commuting model. Among the results, we found that the driving time of a private car in the school commuting phase can be reduced by 23.36%, the dwell time can be reduced by 92.29%, and the driving time in the work and home phase can be reduced by 7.44%. Compared with the school-bus commuting mode, the school-bus time cost of joint commuting can be reduced by 54.88%. In addition, by analyzing the impact of various factors on the objective function and vehicle emissions, it can be seen that staggered commuting to school, regulating regional traffic volume, increasing parking spaces, and improving the utilization of parking spaces can effectively reduce the operating time cost of vehicles and exhaust emissions. The joint-commuting model proposed in this paper considers the balance between service level and resource consumption. While meeting the door-to-door travel needs of students, it can effectively reduce the travel costs of parents and school-bus operation costs, and it can alleviate traffic congestion around schools and reduce the impact on the environment.

## 1. Introduction

Commuting-student travel is an important part of urban-transportation-travel demand. With the improvement of residents’ income levels and the improvement of the urban transportation infrastructure, students’ commuting methods have become diversified. Students’ commuting modes mainly include riding in a private car, cycling, riding a school bus, or riding public transit. He and Giuliano [1] found that students in the Los Angeles area have a high car dependency. More than 60% traveled to school by private car, about 24% on foot or by bicycle, slightly more than 11% by school bus, and only 0.09% by public transport. Yu and Liu [2] conducted a survey of three primary schools in Beijing and found that the proportion of commuting trips by private car reached 46.8%, and the proportion of trips by school bus accounted for 28.9%. Han et al. [3] used stratified-sampling OD survey data to analyze the travel characteristics of primary and secondary school students going to and from school. The results showed that traveling by private car and bicycle dominate the transportation modes in China, accounting for about 60%. Campbell [4] found that parents choose cars to pick up students for commuting, rather than school buses, for convenience and safety. Moreover, due to imbalanced educational resources, the distance between home and school has increased, which makes private-car commuting the preferred choice. All of the previous studies found that the increase in home-to-school distance is the primary reason for the decline in the share of active commuting [5,6]; private-car commuting leads to a high concentration of traffic around schools during commuting periods. In addition, parking facilities at the entrances to schools are imperfect, and traffic organization is unreasonable, resulting in traffic-congestion problems. Chang [7] conducted a survey on several primary schools in Changchun and found that the proportion of commuting by private vehicles reached 27%, and the share of vehicles picking up children on the road in front of the school accounted for 26% of the total vehicles on the road. The traffic volume induced by student transfer increases road congestion, and the average parking delay time for vehicles is 3–4 min on the 457 m road in front of the school gate. Shi [8] analyzed the influence of student-shuttle-vehicle trips on Beijing road traffic. The results showed that the means by which student-shuttle-vehicle trips influence Beijing road traffic include frequent lane-changing during early peak hours and curb-parking during night peak hours, resulting in diminished road capacity. The traffic congestion problems that come with private-vehicle commuting include delays, which generate greater exhaust emissions and increased fuel consumption.

School-bus commuting has become an excellent solution for school travel, considering the alleviation of traffic pressure around the school and the policy of energy conservation and emission reduction. The school-bus routing problem has been of concern and therefore studied for nearly 50 years. Ellegood et al. [9] reviewed 29 articles on the school-bus routing problem (SBRP). Khan et al. [10] studied the influencing factors of students’ choice of commuting mode based on a stated-preference (SP) survey. The results showed that students are more sensitive to the cost of the school bus, the time inside and outside the bus, and comfort when they choose to travel by school bus. Therefore, the objective of school-bus planning is generally to consider the service level of the school bus while reducing the operating costs, including reducing the number of buses, reducing the operating mileage, and minimizing the travel time of the students. In Caceres et al. [11], the primary objective is stated as minimizing the number of buses, followed by implementing improvement heuristics so as to minimize total bus-travel time. Campbell et al. [12] presented a discrete algorithm for finding bus trips, and then employed a trip-linking, assignment-based model minimizing the number of buses used and the travel distance. Shafahi et al. [13] minimized the total cost function, which is related to transit utilization, travel compatibility, and travel time. Bögl et al. [14] adopted a bi-objective function to minimize the sum of the total travel time of the buses and the penalty for exceeding the maximum number of allowed transfers. Riera-Ledesma and Salazar-González [15,16] proposed a bi-objective model to minimize student walking distance and bus-route length. In addition, service quality, fairness, and emissions are other possible objectives. Corberá et al. [17] and Pacheco and Marti [18] considered minimizing total student riding time. Lima et al. [19] proposed a multi-objective model which consisted of the total weighted travel time of the students, the balance of load, and the routing costs. Yan et al. [20] minimized the delay time and waiting time of passengers. Regarding the solution algorithm, as pointed out by Park and Kim [21], SBRP is an NP-hard problem, and for small-scale data sets, exact algorithms can be applied to solve them. Exact algorithms have been proposed, such as Branch and Bound [22], branch-and-cut [23] and column generation [24]. However, more studies are needed so as to solve large problems. Therefore, heuristic algorithms have been developed to quickly obtain satisfactory solutions. Heuristic algorithms that have been used widely to solve SBRP include the saving method [25], the insertion method [26], the sweep algorithm [27], etc. Subsequently, Simulated Annealing (SA) [28], Ant Colony (ACO) [29,30], Tabu Search (TS) [31,32], Genetic Algorithm (GA) [33,34], and GRASP [35] have been widely used to solve school-bus routing problems. 

Despite all of this, the single-school-bus model has many defects in actual operation. For example, a single-school-bus model needs to purchase corresponding vehicles for commuting, which are not highly utilized and cannot meet the door-to-door travel needs of all students. The unstable running time of the school bus leads to long waiting times, with some students spending a long time on the bus, and it is difficult to take care of students who have long journeys. When parents and students commute on the same route, the time and economic cost of parents picking up and dropping off their children would be reduced, so it would be more convenient to choose private vehicles for commuting. Therefore, based on the ideas of “public–private cooperation” and “parking sharing”, combined with the characteristics of the family travel chain during the commuting period, we propose a two-phase commuting model for primary and secondary schools, utilizing both school buses and private cars. In this paper, according to the relevant theories on the school-bus routing problem, a two-phase commuting travel model is constructed based on the single-school and single-vehicle problem. In the first phase, the model is constructed with the minimum time cost of private cars as the optimization objective and solved by genetic algorithm. In the second phase, a model is constructed with the minimum running time of the school buses as the optimization objective, and the Ant Colony algorithm is used to solve the problem. The paper is organized as follows: Section 2 gives a description of the problem and conducts commuting patterns and cost analysis. Section 3 proposes a two-phase, joint-commuting travel model. Section 4 carries out the design of the model-solving algorithm. Section 5 carries out a case analysis. Finally, Section 5 concludes the paper and makes recommendations for future research.

## 2. Problem Statement

### 2.1. Commuting Model Analysis

With the continuous development of urban transportation, the commuting mode of students has gradually diversified, including the slow commuting model, which is based on walking and using non-motorized vehicles; the public transportation model, which is based on using rail transit and buses; and the customized transportation model, which is based on school-bus and private-car commuting. Walking and non-motorized commuting models are suitable for short- and medium-distance travel. Non-motorized vehicle commuting has certain requirements for urban terrain and climate conditions. Rail transit and regular bus commuting depend on the level of the public transportation network and the availability of operating service near schools and in residential areas. The school bus has the characteristics of customized public transportation, which can use transportation resources in a centralized and effective manner, and it has a low occupancy rate of urban-road traffic resources, but it has poor flexibility. Although private-car commuting is more flexible, it has a high occupancy rate of urban-road traffic resources, which can easily cause local, or even large-scale regional, traffic congestion in a city.

Based on the idea of “public–private cooperation”, we attempt to consider both the balance between flexibility and resource occupation, and the association of student commuting with parent commuting in the “family travel chain”, and present the shared parking lot as the transfer station. This paper puts forward a two-phase, joint-commuting model using both private cars and school buses, exploring its advantages and applicability and conducting a modeling analysis. Since the process of students going to and from school is opposite, for simplicity, we only study the process of going to school. This paper involves three models: a private-car commuting model (CM), a school-bus commuting model (SM), and a joint-commuting model (JM). 

We define $P^{+}$ as the set of the transfer stations; school is denoted by $P^{-}=\left\{0\right\}$; *P* is the set of transfer stations and school; *H* is the set of home site (multiple families may exist at the same home site); *D* is the set of work place; *M* is the set of school buses; the number of students at site *I* is $N_{i}$; the number of students picked up by school bus *m* at station *i* is $n_{mi}$; the number of students in the bus is $l_{mi}$; $d_{ij}$ is the distance from *i* to *j*; changes in road traffic conditions are not considered; the average speed of all private cars is $v^{c}$; the average speed of all school buses is $v^{b}$; and the driving time of the vehicle is determined by the average driving speed of the vehicle.

#### 2.1.1. School-Bus Commuting Model

In the process of school-bus commuting, the school bus starts from the parking lot. (In this study, there is no fixed parking lot, and we are ignoring the journey from the parking lot to the first station.) It then runs along the predetermined path, picks up the students at each station along the way, and takes them to the school. In addition, in terms of time, it is also necessary to stipulate that all of the students must arrive at the school within the time window specified by the school, that is, the earliest and latest arrival times as specified by the school. In the process of transportation, the total number of students cannot exceed the capacity *Q* of the school bus at any time, and the riding time of the students cannot exceed the maximum time $T_{\max}$.

**Figure 1:**
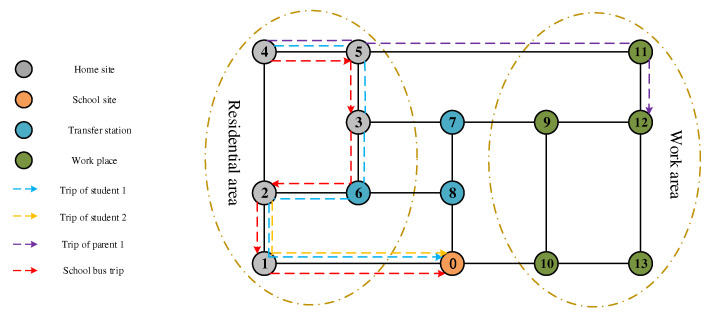
Network diagram of the school-bus commuting model.

School-bus commuting involves the school-bus supplier, the students, and the parents. Considering the “family travel chain”, parents are generally divided into those who go to work and those who do not. Taking Figure 1 as an example, student 1 living at home site 4 takes a school bus from home site 4 to school station 0; student 2 living at home site 2 takes the school bus from home site 2 to school 0. The school bus picks up students along the 4–5–3–2–1 station route, and finally arrives at school station 0. At the same time, parents who need to work go directly to the workplace. For example, parent 1, who lives at home site 4, takes a private car to their corresponding workplace 12, and the travel chain is 4–12; parents who do not need to go to work do not travel (e.g., parent 2 living at home site 2).

#### 2.1.2. Private-Car Commuting Model

**Figure 2:**
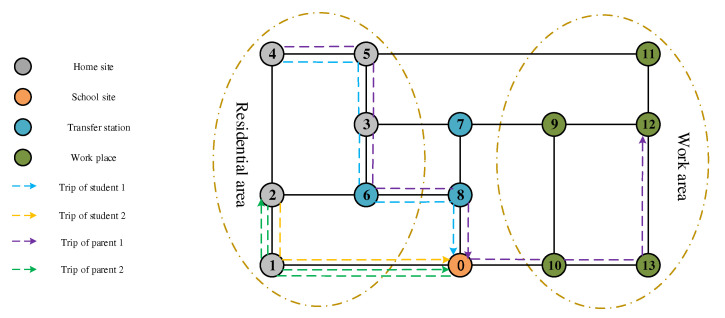
Network diagram of the private-car commuting model.

Private-car commuting only involves family groups, considering the “family travel chain”, in the process of traveling to school; parents who have work needs will drive their children to school and then go to work in private cars. For parents who do not work, they will drive their children to school and return home in a private car. Taking Figure 2 as an example, family 1 living at home site 4 and family 2 living at home site 2 each take a private car to school 0, stop at the school site, and the students disembark; the travel chain of student 1 is 4–0, and that of student 2 is 2–0. Afterwards, parent 1, who has work needs, goes to workplace 12, and the travel chain is 4–0–12. Parent 2, who does not need to go to work, returns to the home site 2, and the travel chain is 2–0–2.

#### 2.1.3. Joint-Commuting Model

In the proposed joint-commuting model, parents would first use private cars to take students to the transfer station, go to work or return home, and then the school bus would pick up the students from each transfer station and take them to school. The transfer station would be a place that has free parking resources that are easy to share during the period when students travel to and from school. It would also have certain requirements for the convenience of vehicle access and the security of primary and secondary school students. For the selection of transfer stations, this paper considers government agencies and state-owned enterprises with idle parking spaces that are close enough to the school such that the students would arrive on time. The transfer station would only be used as a temporary turnover place for students. Since students go to school before the working hours of government agencies and state-owned enterprises begin, such use would not affect the normal use of government agencies or enterprises during the working period. The use of the parking lot of the transfer station would free of charge with the consent of the government agencies and enterprises manager.

In the first phase of the joint-commuting model, the transfer stations would be selected according to the travel costs of the parents, and parents would use private cars to take their students to the transfer stations and then go to their workplaces. In the second phase, according to the number of students at the transfer station obtained in the first phase, the bus operation cost would be considered, and the bus stops would be selected from the transfer stations to plan the bus route. *M* school buses with the same capacity *Q* would depart from the school and transfer students to the school via the transfer stations according to the designated routes. If the students at a certain station could not all be served by the same school bus due to the insufficient passenger capacity of the vehicles, the remaining students would be picked up and dropped off by additional school buses.

**Figure 3:**
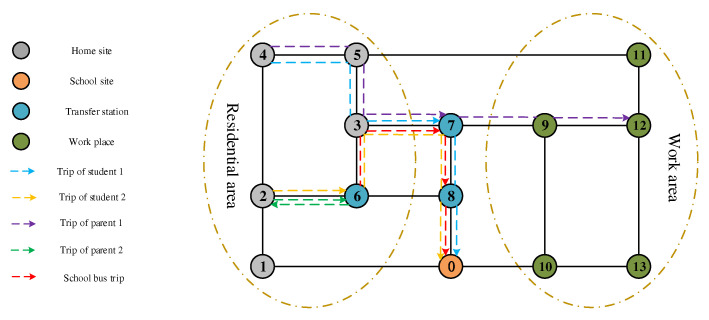
Network diagram of the joint-commuting model.

Joint commuting involves school-bus suppliers, students, and parents. Taking Figure 3 as an example, family 1 living at home site 4 goes to transfer station 7 by private car; family 2 living at home site 2 goes to transfer station 6 by private car, stops at the connecting station where students disembark, and then parent 1, who needs to work, goes to work place 12, and the travel chain is 4–7–12; parent 2, who does not need to do to work, returns to home site 2, and the travel chain is 2–6–2. After the students arrive at the corresponding transfer stations, the school bus will pass through transfer stations 6–7–8 to pick up the students, and finally they will reach school 0 and drop the students off; the travel chain for student 1 is 4–7–0, and that for student 2 is 2–6–0.

### 2.2. Commuting Cost Analysis

This paper mainly considers the travel time cost of each model from the perspective of transportation vehicles, which consists of the driving and dwell time of the school buses (SB) and the time of the private cars (PC). Among the individual travel costs, the driving and dwell time costs of the private cars are regarded as the travel costs for the parents; the student travel cost is related to the driving and dwell time of the private cars, the driving and dwell time of school buses, and the waiting time, which are reflected through relevant constraints in the following text.

#### 2.2.1. School-Bus Commuting Costs

For the cost of the school bus, we do not consider the purchase cost or maintenance costs of the vehicle, and the operation cost takes into account the school-bus driving time and dwell time; the driving time cost of the school bus in the operation process is $t_{ii^{\prime }}^{b}=d_{ii^{\prime }}/v^{b}$; the cost of getting on and off the bus is incurred when the school bus arrives at the pick-up and drop-off sites. The service time for bus *M* after it arrives at stop *i* is related to the number of students getting on or off the bus at that stop. According to the regression model developed by Brace et al. [36], the boarding service time is $T_{mi}^{f}=19+2.6\cdot n_{mi}$, the service time for getting off is $T_{mi}^{f}=29+1.9\cdot n_{mi}$. The total cost of school-bus service is $T^{b}=t_{ii^{\prime }}^{b}+T_{mi}^{f}$. The travel cost $T^{c}$ of a private car is the travel time cost $t_{ik}^{c}=d_{ik}/v^{c}$ for parents to travel from home to work; the cost of parents without work needs is 0, so $T^{c}=t_{ik}^{c}$. The student travel cost includes the student’s walking time, waiting time, and ride time, which is considered in this paper according to the constraints.

#### 2.2.2. Private-Car Commuting Cost

In the case of private-car commuting, the costs include travel time $t_{i0}^{c}$ during the school commute phase, dwell time $T_{0}^{c}$ at a school stop, and driving time $t_{0k}^{c}$ during the work or home phase. The drop-off process of private cars at school sites can be regarded as a multi-service desk queueing model (M/M/s/∞) [37,38]. Assuming that the time interval between successive arrivals of vehicles conforms to the negative exponential distribution of the parameter, the drop-off area is regarded as multiple service desks; the number of service desks is determined by the number of drop-off parking spaces s; and the service time of each service desk obeys the negative exponential distribution of parameter μ; the drop-off process is the service process.

Due to the limitation of the school-bell time, all students are required to arrive within the specified time period Δt, and the arrival rate is $\lambda =N_{0}/\Delta t$.

The service rate μ depends on the drop-off time of the vehicle $t_{u}$ and the time when the vehicle starts to merge into the traffic lane $t_{v}$:

(1) $$\mu =1/\left(t_{u}+t_{v}\right)$$

According to the gap theory [39,40], the maximum flow into which a drop-off vehicle can pass into the roadway is equal to:

(2) $$q_{d\max}=\frac{qe^{-q\tau }}{1-e^{-qh}}$$

where $q_{d\max}$ is the maximum flow of drop-off vehicles into the traffic lane ($veh\cdot s^{-1}$), q is the vehicle flow rate in the roadway ($veh\cdot s^{-1}$), τ is the critical neutral time interval at which the drop-off vehicle can merge into the roadway (s), and h is the headway when the drop-off vehicles entering the lane continue to follow (s).

The time when the vehicle starts to merge into the traffic lane can be expressed as: $t_{v}=1/q_{d\max}$.

Then, μ can be expressed as:

(3) $$\mu =1/\left(t_{u}+t_{v}\right)=1/\left(t_{u}+1/q_{d\max}\right)=1/\left(t_{u}+\frac{1-e^{-qh}}{qe^{-q\tau }}\right)$$

when $\rho_{s}=\frac{\rho }{s}=\frac{\lambda }{s\mu }<1$, the idle probability can be expressed as:

(4) $$p_{0}={\left[\sum_{n=0}^{s-1}\frac{\rho^{n}}{n!}+\frac{\rho^{s}}{s!\left(1-\rho_{s}\right)}\right]}^{-1}$$

The average waiting time for drop-off vehicles can be calculated as:

(5) $$W_{s}=\frac{L_{s}}{\lambda }=\frac{L_{q}+\rho }{\lambda }=\frac{c\left(s,\rho \right)\rho_{s}}{1-\rho_{s}}=\frac{\rho^{s}p_{0}\rho_{s}}{s!{\left(1-\rho_{s}\right)}^{2}}$$

The average waiting time for private cars to drop off students at the school station is recorded as $W_{0s}$; then, the dwell time at the school station $T_{0}^{c}=W_{0s}$. The total cost of the parent is equal to the cost of the private car, $T^{c}=t_{i0}^{c}+T_{0}^{c}+t_{0k}^{c}$. Student travel cost is the sum of driving time during the school commute and drop-off time at the school station.

**Figure 4:**
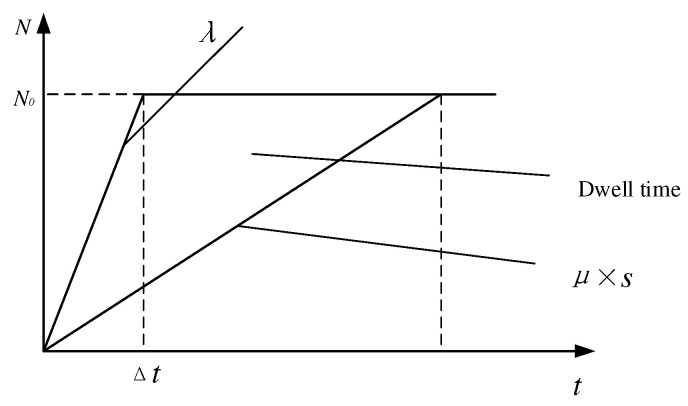
The arrival and departure curves of private cars at the school site in the case of $\rho_{s}>1$.

When $\rho_{s}>1$, queuing theory is not applicable to calculate the average waiting time for drop-off vehicles at stations; the dwell time of all private cars at the school site can be obtained according to the relationship between family arrivals and departures in Figure 4, and then the average waiting time can be obtained:

(6) $$T_{0}^{c}=\frac{\int_{0}^{\Delta t}\left(\lambda -\mu \cdot s\right)+\int_{\Delta t}^{N_{0}/\left(\mu \cdot s\right)}\left(N_{0}-\mu \cdot s\right)}{N_{0}}$$

#### 2.2.3. Joint-Commuting Cost

The total cost of the school bus is calculated by the sum of the service time for pick-up and drop-off at the transfer stations and the school, plus the driving time, which can be expressed as:

(7) $$T^{b}=t_{jj^{\prime }}^{b}+T_{mj}^{f}$$

The total cost of parents is equal to the cost of the private cars, which consist of the driving time in the school commuting phase and the parking time at the transfer station, along with the driving cost in the work phase or in the home phase. It can be expressed as:

(8) $$T^{c}=t_{ij}^{c}+T_{j}^{c}+t_{jk}^{c}+t_{ji}^{c}$$

Similarly, $T_{j}^{c}=W_{js}$.

The student’s travel cost is the sum of time to the transfer station, drop-off time at the transfer station, and time on the school bus.

## 3. Proposed Model

A two-phase, joint-commuting model is proposed in this paper. In the first phase, the objectives are to minimize the time cost incurred by private cars driving to school, the workplace, and home, and to formulate a plan for transfer-station selection and vehicle-parking allocation. The second phase aims to minimize school-bus operation time and to formulate the school-bus route.

### 3.1. Model Parameters

The parameters and decision variables in the mathematical model are shown in Table 1.

### 3.2. Private-Car Travel Phase

Considering the travel intention of the parents in family *a*, in order to increase the convenience of the parents, the objective of the first phase is to minimize the cost of private-car travel in the joint-commuting model. According to the cost analysis, the driving time of private cars during the school commute phase is expressed as $\sum_{a\in A}\sum_{i\in H}\sum_{j\in P}x_{aij}t_{aij}^{c}$, the driving time of the home phase is expressed as $\sum_{a\in A}\sum_{i\in H}\sum_{j\in P}x_{aij}X_{aij}t_{aji}^{c}$, the driving time of the work phase is expressed as $\sum_{a\in A}\sum_{i\in H}\sum_{j\in P}\sum_{k\in K}x_{aij}X_{aijk}t_{ajk}^{c}$, and the dwell time at the transfer station is expressed as $\sum_{a\in A}\sum_{j\in P}\left(T_{aj}^{c}\sum_{i\in H}x_{aij}\right)$.

The mathematical model aimed at minimizing the total cost of private-car travel is expressed as:

(9) $$\min F_{1}=\sum_{a\in A}\sum_{i\in H}\sum_{j\in P}x_{aij}t_{aij}^{c}+\sum_{a\in A}\sum_{i\in H}\sum_{j\in P}x_{aij}X_{aij}t_{aji}^{c}+\sum_{a\in A}\sum_{i\in H}\sum_{j\in P}\sum_{k\in K}x_{aij}X_{aijk}t_{ajk}^{c}+\sum_{a\in A}\sum_{j\in P}\left(T_{aj}^{c}\sum_{i\in H}x_{aij}\right)$$

Subject to:

(10) $$\sum x_{aij}=1,\forall a\in A,\forall i\in H,\forall j\in P$$

(11) $$\sum_{i\in H}x_{aij}=\sum_{i\in H}X_{aiji}+\sum_{k\in D}X_{aijk},\forall a\in A$$

Formula (9) is the objective function, constraint (10) requires that family *a* must and can only choose one transfer station; constraint (11) ensures that parent *a* chooses to work or go home after visiting the transfer station.

### 3.3. School-Bus Travel Phase

The majority of the papers on the sub-problem of bus-route generation in SBRP try to improve the efficiency of route planning by minimizing the cost, which includes reducing fixed costs by minimizing the number of buses and reducing variable costs by minimizing the total trips or total time [9]. From the perspective of school-bus operators, the optimization objective is to minimize school-bus costs. The purchase cost and maintenance costs for the bus are not considered here, and the school-bus cost only reflects the variable cost related to the operating time. According to the cost analysis, the driving time of the school bus is the driving time from the first station to the school, which can be expressed as $\sum_{m\in M}\sum_{j\in P^{+}}\sum_{j^{\prime }\in P}t_{jj^{\prime }}^{b}y_{jj^{\prime }}$, the dwell service time of the school bus at the transfer station can be expressed as $\sum_{m\in M}\sum_{j\in P}T_{mj}^{f}$.

The mathematical model aimed at minimizing the school-bus cost [14,15,16,20,41] can be formulated as follows:

(12) $$\min F_{2}=\sum_{m\in M}\sum_{j\in P^{+}}\sum_{j^{\prime }\in P}t_{jj^{\prime }}^{b}y_{jj^{\prime }}+\sum_{m\in M}\sum_{j\in P}T_{mj}^{f}$$

Subject to:

(13) $$\sum_{m\in M}\sum_{i\in P}y_{jj^{\prime }m}\geq 1,\forall j\in P^{+}$$

(14) $$\sum_{m\in M}\sum_{i\in P}y_{jlm}-\sum_{m\in M}\sum_{j\in P}y_{lj^{\prime }m}=0,\forall l\in P^{+}$$

(15) $$l_{mj}\leq Q,\forall j\in P^{+}$$

(16) $$l_{mj}+n_{j^{\prime }}-l_{mj^{\prime }}\leq M_{1}(1-y_{jj^{\prime }m}),\forall j\in P,\forall j^{\prime }\in P^{+}$$

(17) $$Z_{jm}+t_{jj^{\prime }}^{b}+T_{mj^{\prime }}^{f}-Z_{j^{\prime }k}\leq M_{2}(1-y_{jj^{\prime }m}),\forall j\in P,\forall j^{\prime }\in P^{+}$$

(18) $$l_{m0}=0,\forall m\in M$$

(19) $$Z_{jm}\leq T_{\max},\forall j\in P^{+}$$

(20) $$\sum_{m\in M}n_{mj}=N_{j},j\in P^{+}$$

Formula (12) is the objective function, and the constraint (13) requires that every station must be visited by a school bus and be visited at least once; constraint (14) requires that the school bus must leave after visiting the student site; constraint (15) requires that the school bus must not be overloaded after visiting the stop; and constraint (16) represents the change in the number of bus loads at consecutive stops on the bus route; if school bus *m* visits station *j* immediately after visiting station $j^{\prime }$ ($y_{jj^{\prime }m}=1$), the school-bus capacity satisfies $l_{mj}+n_{j^{\prime }}=l_{mj^{\prime }}$, when $y_{jj^{\prime }m}=0$, $l_{mj}$ has no relation with $l_{mj^{\prime }}$, and a sufficiently large positive integer *M*_1_ is further introduced to transform the nonlinear inequality into a linear inequality. Similarly, constraint (17) represents the relationship between the school-bus travel time of continuous stations in the school-bus path, and a sufficiently large positive integer *M*_2_ is introduced to transform the nonlinear inequality into a linear inequality. Constraint (18) indicates that the school bus leaves the school without students, constraint (19) requires that the travel time of students does not exceed the maximum travel time $T_{\max}$, and constraint (20) ensures that all students are picked up.

## 4. Solution Methods

We solve the joint-commuting model in two stages. The first stage: take the private-car travel route as the optimization objective, obtain the transfer station selected by each parent and the number of students to be picked up at each transfer station under the condition of the minimum total cost. Due to the limitation of site capacity, it is difficult to meet the needs of each parent. In order to ensure fairness, parents’ behavioral choices are not considered here, consider the overall efficiency of the system, and deal with this model as an assignment problem. Since the transfer station selection scheme is a nonlinear programming problem with 0-1 decision variables, a genetic algorithm is a common tool for solving nonlinear programming problems, so a genetic algorithm is selected to solve.

In terms of chromosome coding of a genetic algorithm, a chromosome is composed of 0 and 1; each gene is a binary variable. Each gene indicates whether the alternative transfer station is selected; “1” means that the transfer station (or school) is selected, and “0” means that the transfer station (or school) is not selected. The solving steps of a genetic algorithm are as follows:

Step 1 Parameter initialization: set the number of iterations to 1000, the crossover probability to 0.7, the mutation probability to 0.001, the selection rate to 0.5, and the population size to 100;

Step 2 Input initial data and related parameters. The initial data include the distance matrix between each station, the number of drop-off parking spaces, and the vehicle flow rate at the transfer station, τ, h, Δt, $t_{u}$;

Step 3 An initial population is randomly generated, and its chromosomes contain the initial transfer station selection scheme;

Step 4 Calculate chromosomal fitness. Since the objective function of the model is a minimization problem, and the value is always positive, the fitness function is expressed by the reciprocal of the objective function value. Assuming that the objective function value of an individual *o* is g(o), the fitness value corresponding to the chromosome is z(o)=1/g(o), and for individuals that do not meet the constraints, the fitness value is 0;

Step 5 Select operator. Individuals with high fitness in the population can be directly reproduced as parent chromosomes for operation selection, while other chromosomes are operated and selected in the way of roulette.

The probability that an individual *o* is selected is $P_{o}=\frac{z\left(o\right)}{\sum z\left(o\right)}$.

Where $P_{o}$ represents the probability that the individual is selected, z(o) represents the chromosome fitness, o represents the individual in the population;

Step 6 Cross and mutate chromosomes.

Using the adaptive crossover probability, the crossover probability will be automatically adjusted with the different fitness function values during the calculation process. The specific formula is:

(21) $$P_{c}=\{\begin{array}{c} \frac{K_{1}\left(z_{\max}-z\right)}{z_{\max}-z_{avg}},z\geq z_{avg}\\ K_{2},z<z_{avg} \end{array}$$

where $P_{c}$ is the adaptive crossover probability, $z_{\max}$ is the maximum fitness function value of the individual in the group, $z_{avg}$ is the average fitness function value of each generation of the group, z is the two individuals that generate crossover, one of which is the larger fitness function value, $K_{1}$, and $K_{2}$ are constants in the interval (0, 1).

Using adaptive mutation probability:

(22) $$P_{m}=\{\begin{array}{c} \frac{K_{3}\left(z_{\max}-z\right)}{z_{\max}-z_{avg}},z\geq z_{avg}\\ K_{4},z<z_{avg} \end{array}$$

where $P_{m}$ is the adaptive mutation probability, $K_{3}$, $K_{4}$ are constants in the interval (0, 1);

Step 7 Judge the fitness function value. After completing Step 6, jump to Step 4, recalculate the fitness function value, make a judgment, and then continue the loop solution;

Step 8 Determine whether the number of iterations has been reached. Return to Step 5 if the maximum number of iterations has not been reached; if the number of iterations has been reached, terminate the algorithm, and output the location selection scheme.

The second stage: for the route planning problem of multiple buses, the transfer stations are first grouped, and each group can be transformed into a separate TSP route optimization problem.

**Figure 5:**
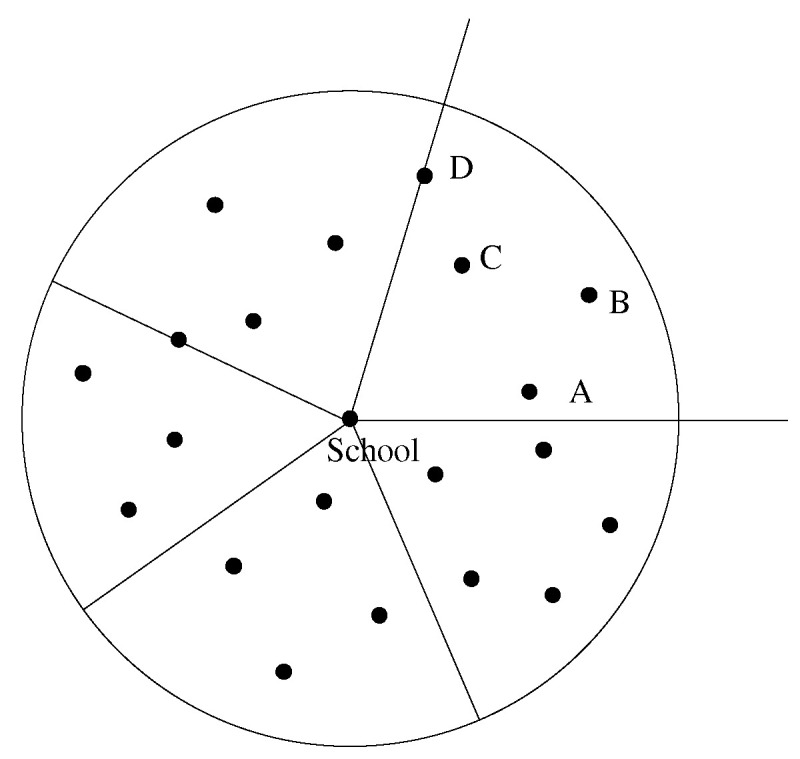
Site grouping strategy. (The dots represents transfer stations, and the stations in the fan-shaped area formed by two adjacent letters are a group).

The solution steps are as follows:

Step 1 Group the transfer stations. As shown in Figure 5, with the school as the center of the area, the initial position of the ray, starting from the school, does not intersect with any station position, and it moves counterclockwise to transfer station B to judge whether the number of students exceeds the rated capacity of the school bus. If not, all of the students at this station will be served by the bus, while the number of people on the bus will be added up, and the next transfer station, C, will be found. When the number of students at connecting station D exceeds the rated passenger capacity of the school bus, the student demand at connecting station D will be split, and the sector area formed at this time is divided into a group. Meanwhile, the number of remaining students at connecting station D is calculated and divided into the next area, and so, on until all sites have been divided.

Step 2 Use the Ant Colony algorithm to find the optimal school-bus route scheme for each group.

Step 2.1 Initialization parameters: read the interlinking site data of each partition, and set the initial parameters of the number of ants e, the number of iterations, the pheromone factor α, the heuristic function factor β, the pheromone constant $Q^{*}$, and the pheromone volatility factor $\rho^{*}$;

Step 2.2 Construct the solution space:

Assuming that the ants leave the school, the initial point of the ant is defined as 0 for each ant w (w=1,2,⋯,e); the following probability transfer formula is obtained according to the roulette method to calculate the next connecting station to be visited; transfer the ant to the selected visiting station; and add the re-selected point to the tabu table and route record table until all nodes in the tabu list are visited, and the current path length is obtained.

The transition probability formula is:

(23) $$P_{jj^{\prime }}^{w}=\{\begin{array}{c} \frac{{\left[\tau_{jj^{\prime }}^{*}(t)\right]}^{\alpha }\cdot {\left[\eta_{jj^{\prime }}(t)\right]}^{\beta }}{\underset{g\in {\mathrm{allow}}_{w}}{\sum }{\left[\tau_{jg}^{*}(t)\right]}^{\alpha }\cdot {\left[\eta_{jg}(t)\right]}^{\beta }},g\in {\mathrm{allow}}_{w}\\ 0,g\notin {\mathrm{allow}}_{w} \end{array}$$

where $P_{jj^{\prime }}^{w}$ represents the transition probability of the ant w at the transfer station j and transfer station $j^{\prime }$ at time t; $\tau_{jj^{\prime }}^{*}$ represents the concentration of pheromone remaining on the line between transfer station j and transfer station $j^{\prime }$ at time t; at the initial moment, the amount of information on each line is the same; and let $\tau_{jj^{\prime }}^{*}\left(0\right)=C$ (*C* be a constant); α represents the heuristic factor of the residual information amount on the line; β represents the heuristic factor of the expected value; $\eta_{jj^{\prime }}$ represents the expected value of the ant from station j to station $j^{\prime }$; and $allow_{w}$ represents the ant w path not yet traveled.

For the initially set *e* ants, repeat the above process to complete an optimization process. Compare the iterative optimization results of *e* ants, and write down the optimal running route;

Step 2.3 Update pheromone:

According to the pheromone update rule, update the pheromone and clear the tabu table and program record table.

The updated formula of the pheromone concentration in the ant system is:

(24) $$\tau_{jj^{\prime }}^{*}\left(t+r\right)=\rho^{*}\cdot \tau_{jj^{\prime }}^{*}\left(t\right)+\left(1-\rho^{*}\right)\cdot \Delta \tau_{jj^{\prime }}^{*},0\leq \rho^{*}<1$$

where $\tau_{jj^{\prime }}^{*}\left(t+r\right)$ represents the pheromone after completing *r* cycles; *r* represents the number of connecting stations on the line; $\Delta \tau_{jj^{\prime }}^{*}$ represents the total amount of pheromone left by all the ants on paths j to $j^{\prime }$ after traversing all the stations, which is:

(25) $$\Delta \tau_{jj^{\prime }}^{*}=\sum_{w=1}^{e}\Delta \tau_{jj^{\prime }}^{*w}$$

where $\Delta \tau_{jj^{\prime }}^{*w}$ represents the amount of pheromone left by the *w*th ant on the path j to $j^{\prime }$. If the *w*th ant goes through the path j to $j^{\prime }$, then:

(26) $$\Delta \tau_{jj^{\prime }}^{*w}=Q^{*}/L^{w}$$

where $L^{w}$ is the total length of the path that the ants have traveled. Otherwise, the pheromone left by the *w*th ant on j to $j^{\prime }$ is 0.

The operating cost of the school bus is the core issue that affects the optimization of the school-bus routes; improving the update operator of the pheromone can effectively select the best route. Combined with the objective function of the school-bus route-planning model, the linear equation of the time weight model is established:

(27) $$\omega_{jj^{\prime }}=\theta y_{jj^{\prime }}^{t}(t_{jj^{\prime }}^{b}+T_{j^{\prime }}^{f})$$

where $\omega_{jj^{\prime }}$ is the time weight from j to $j^{\prime }$; θ represents the trade-off coefficient of the time value; and θ=1.

Introducing the time function f(t) between stations, after completing one cycle, the update operator of the pheromone is as follows:

(28) $$\tau_{jj^{\prime }}^{*}\left(t+1\right)=\left(1-\rho^{*}\right)\cdot \tau_{jj^{\prime }}^{*}(t)+f\left(t\right)$$

(29) $$f\left(t\right)=\sum \sum y_{jj^{\prime }}^{t}t_{jj^{\prime }}^{b}$$

For the school bus, improving the pheromone update operator will increase the pheromone of the least time-consuming path, which will lead more ants to choose.

Step 2.4 Determine whether the set number of iterations has been reached. If it has not reached the maximum number of iterations, return to Step 2.2; if reached, terminate the algorithm.

Step 3 Repeat Step 2 until all groups complete the route optimization.

**Figure 6:**
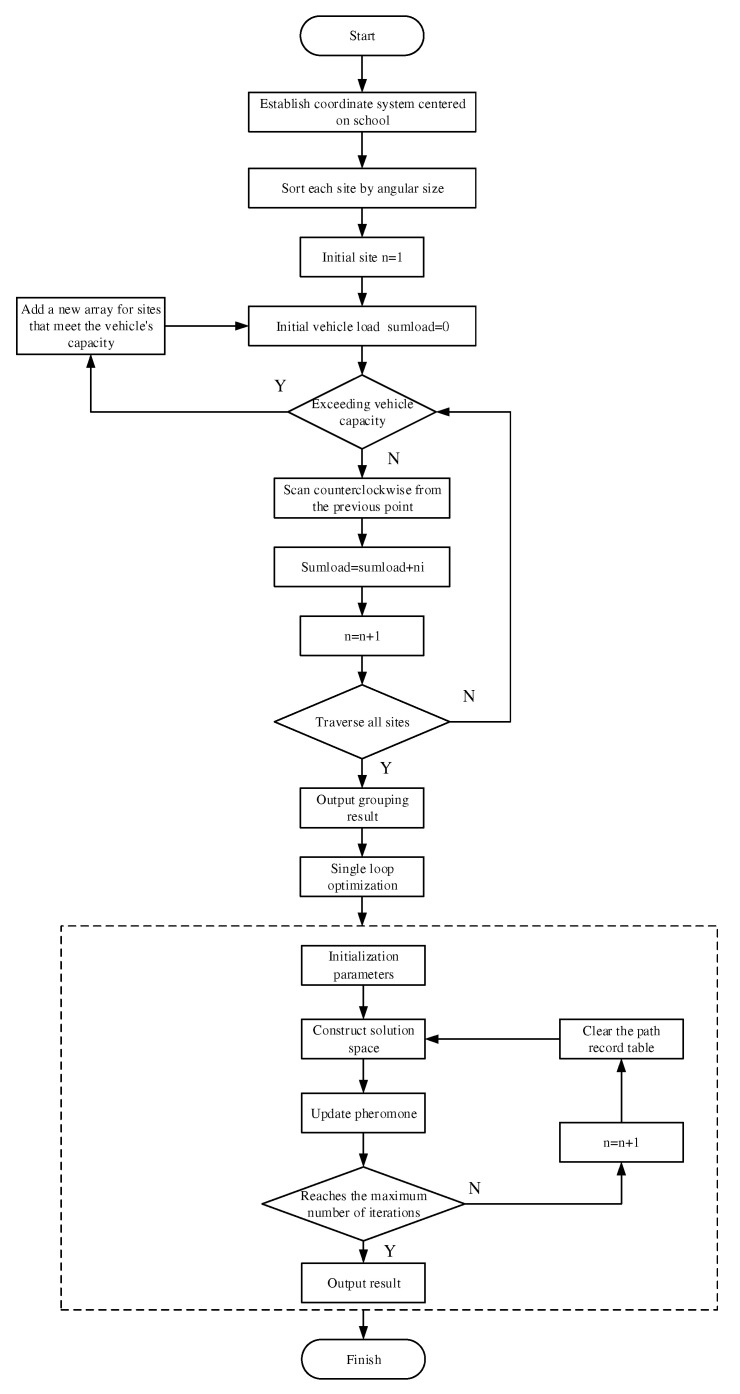
The second stage solution algorithm.

The flow chart of the second stage solution algorithm is shown in Figure 6.

## 5. Case Analysis

### 5.1. Case Description

**Figure 7:**
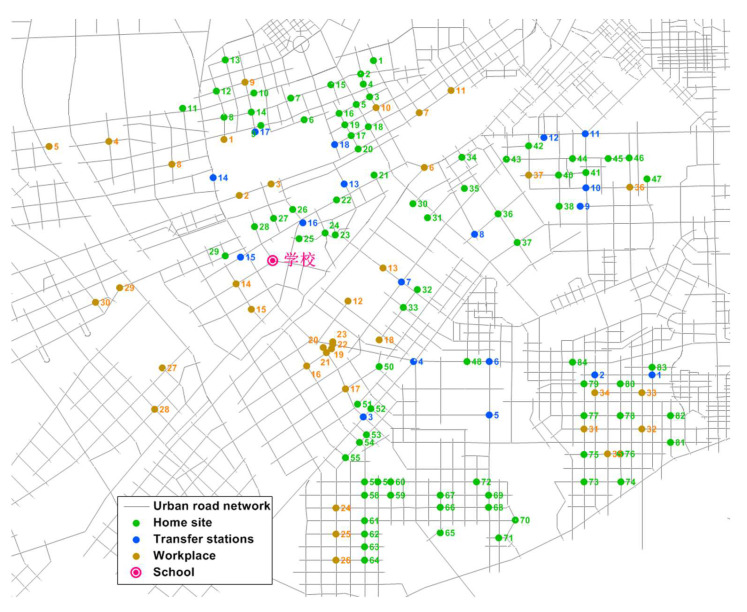
Site location.

We selected a school in Changchun as an example, extracted part of the information of the residents’ travel survey, and selected 252 families who commute by private car and whose work locations are clustered into 37 groups. According to the actual situation around the school, 19 alternative transfer stations for shared parking were selected, and which are shown in Figure 7. We selected 5 school buses, with a capacity of 52 people, and set the average speed of the school bus at 20 km/h. The work of [42] proposes a vehicle-delay model of an urban-road drop-off area and used VISSIM simulation software and actual survey data to calibrate the model parameters, which are consistent with the scenario studied in this paper. Therefore, according to the results of reference [42], take τ = 3.75 s, h = 2.65 s, and the relevant parameters are presented in Table 2.

### 5.2. Result Analysis

**Figure 8:**
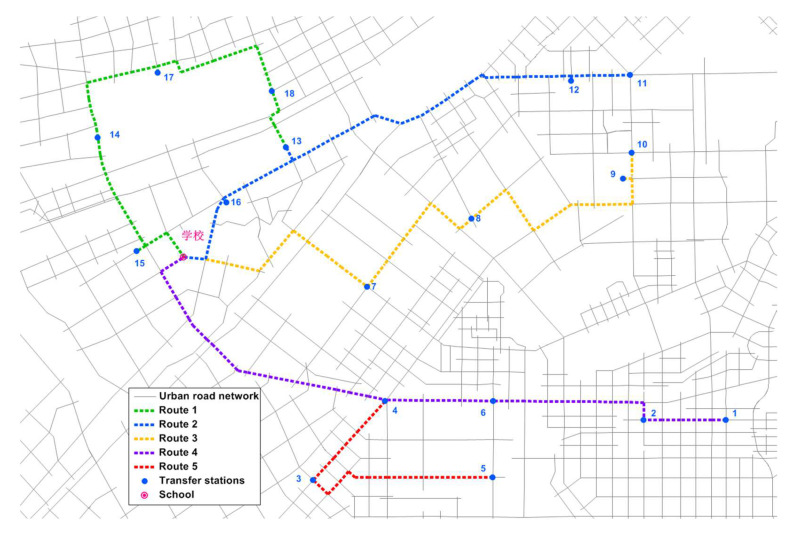
School-bus route.

MATLAB R2021a programming was used to solve the problem, and the genetic algorithm was used to obtain the number of students at the transfer station; the total cost function of private cars was obtained as 101.40 h, and the average travel time cost per parent was 0.4 h; the detailed results are shown in Table 3. The school-bus route solution, based on the number of students at each transfer station, is shown in Table 4. The route of the school bus is shown in Figure 8.

Table 5 and Table 6 show the various costs and comparative data of the school-bus, private-car, and joint-commuting models. It can be seen from Table 5 that the cost of private-car commuting is the highest, followed by joint commuting, and the cost of school-bus commuting is the lowest. The joint-commuting model reduces the total cost by 23.33%, compared with private-car commuting. In the private-car commuting model, the average travel time of parents is 32.26 min, and the average travel time of parents in the joint-commuting model is 24.14 min, which is a decrease of 25.15%. Among these elements, the dwell time and driving time of private cars in the school commute phase decreased by 92.29% and 23.36% respectively, and the driving time in the work or home phase decreased by 7.44%. The result implies that the joint-commuting model solves the problem of traffic congestion around the school, reduces the waiting time of parents at the school, and simultaneously alleviates the detour problem of parents in the case of the “family travel chain”, making parents’ travel routes more reasonable and saving travel time. Although the total cost of the joint-commuting model is higher compared with the school-bus commuting model, due to the “win–win” situation caused by “public–private cooperation”, the travel time of the school bus is reduced by 49.09%.

### 5.3. Sensitivity Analysis

In this section, we select the main factors that affect the system cost for sensitivity analysis, analyze their impact on the system cost and the environment, and explore the applicability of the model proposed in this paper.

Vehicle exhaust emissions are the main cause of environmental pollution; the exhaust pollutants emitted by motor vehicles mainly include CO, HC, and NO; the formula for calculating the exhaust emissions coefficient for a motor vehicle in the driving state of the road section is:

(30) EF=BEF×φ×γ×δ

where EF is the emission coefficient; BEF is the comprehensive benchmark emission coefficient; φ is the environmental correction factor; γ is the road traffic condition correction factor, and δ is the deterioration correction factor; the exhaust emission parameters of cars and school buses are shown in Table 7.

The emission parameters of cars in an idle state in [43] are used as the parking emission coefficient at the station, and the emission parameters of buses in an idle state in [44] are used as the parking emission coefficient of school buses at the station, both of which can be obtained in Table 8.

#### 5.3.1. Sensitivity of the Student Arrival Rate

**Figure 9:**
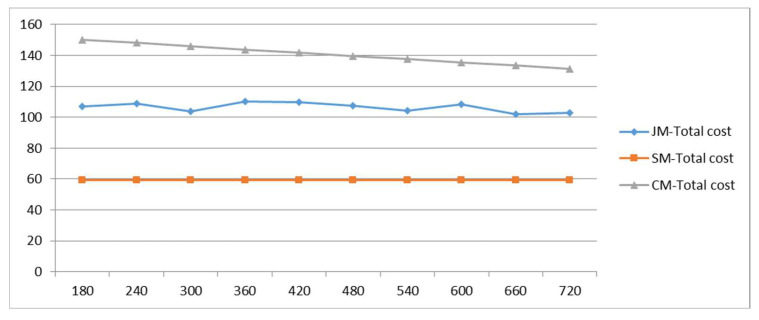
Total cost variations with parameter Δt.

Under the condition that the number of students remains unchanged, the time period of students’ arrival is changed to affect the student arrival rate, and the changes in various costs are analyzed so as to explore the impact of the staggered commute to school on the model. The results are shown in Figure 9; as the arrival time period increases, the student arrival rate decreases, and the cost of private-car commuting decreases. Due to the randomness of the algorithm, the cost of the joint-commuting model fluctuates within a small range and is basically not affected by the variation of the arrival rate, which demonstrates the stability of the model. The school-bus commuting costs remain the same because the size of the student scale remains the same. Within a given range of variation, private-car commuting costs are always higher than joint-commuting costs; joint-commuting costs are consistently higher than school-bus commuting costs; when Δt = 1500 s, the cost of private-car commuting is equal to the cost of joint commuting, and joint commuting loses its advantage.

**Figure 10:**
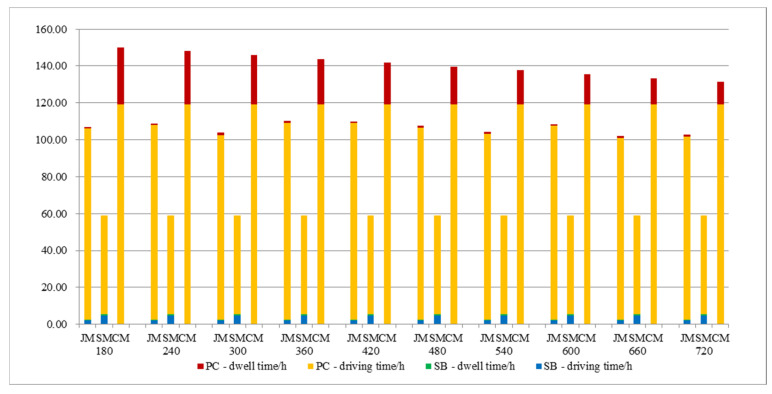
Various cost variations with parameter Δt.

It can be seen from Figure 10 that the travel time of the three commuting models has nothing to do with the arrival rate; the dwell time of private-car commuting decreases with the decrease in arrival rate; the dwell time of school-bus commuting and joint commuting remain relatively stable; and they are always smaller than the dwell time of private-car commuting.

**Figure 11:**
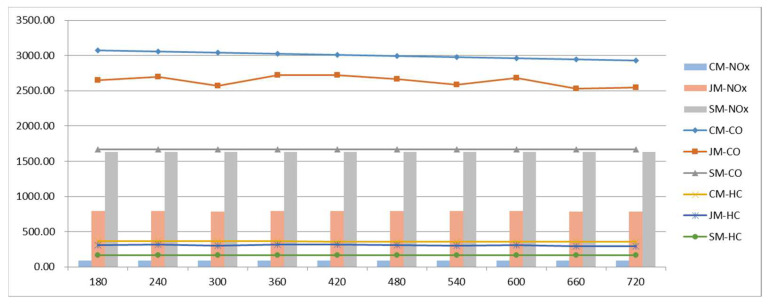
Pollutant emissions variations with parameter Δt.

Figure 11 shows the emission data of vehicle pollutants under the three commuting models. The data show that the two types of CO and HC emissions are ranked from high to low: private-car commuting, joint commuting, and school-bus commuting. The ranking of NO_x_ emissions from high to low is school-bus commuting, joint commuting, and private-car commuting. Emissions from private-car commuting decrease as the arrival time period increases.

In conclusion, the staggered commute-to-school measures can effectively reduce the commuting cost of private cars and reduce vehicle emissions, and they have no significant impact on the school-bus and joint-commuting models.

#### 5.3.2. Sensitivity of Vehicle Flow Rate in Roadway q

**Figure 12:**
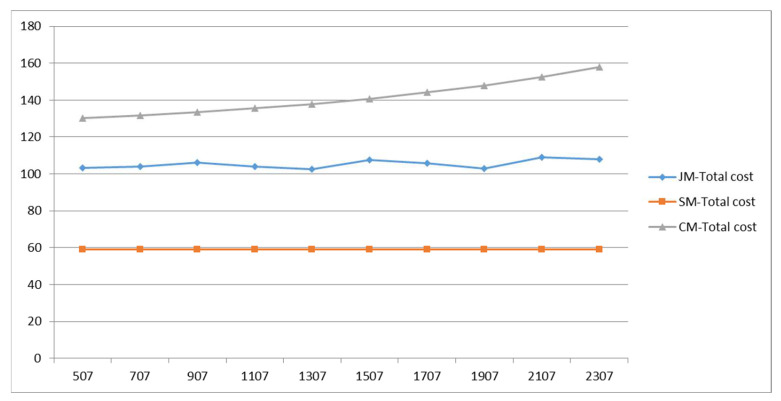
Total cost variations with parameter q.

**Figure 13:**
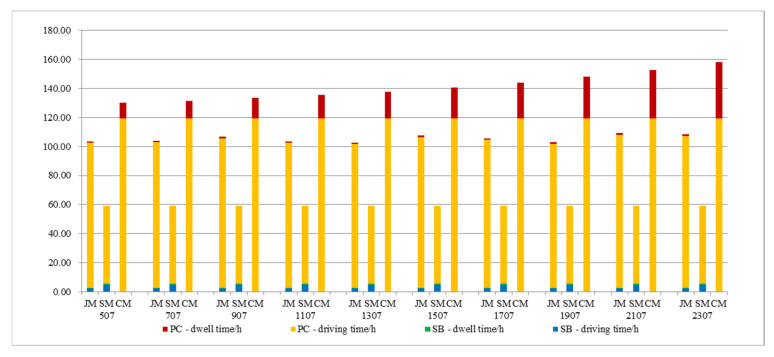
Various cost variations with parameter q.

Vehicle flow rate in roadway q affects the time when private cars leave the parking space; the variation of each cost with q is analyzed to explore the impact of regional traffic control optimization on the model. The results are shown in Figure 12; with the increase of q at the school entrance, the cost of private-car commuting increases; due to the randomness of the algorithm, the cost of the joint-commuting model fluctuates within a small range and is basically not affected by the variation of q, which demonstrate the stability of the model. The school-bus commute costs remain the same because the size of the student scale remains the same. The cost variation results of the three commuting models are shown in Figure 13. As can be seen from the figure, the dwell time of private-car commuting increases rapidly with the increase of q; the other costs remain basically unchanged except for the small fluctuation of the school-bus driving time in the joint-commuting model.

**Figure 14:**
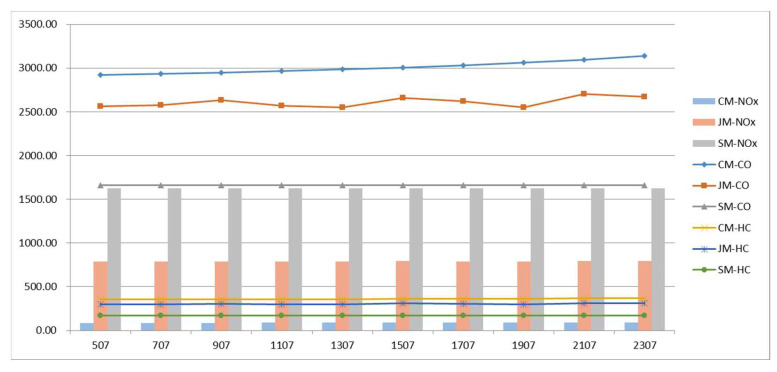
Pollutant emissions variations with parameter q.

Figure 14 shows vehicle emissions data for the three commuting models. The data show the two types of CO and HC emissions as ranked from high to low: private-car commuting, joint commuting, and school-bus commuting. The ranking of NO_x_ emissions from high to low is school-bus commuting, joint commuting, and private-car commuting. As vehicle flow rate in the roadway increases, emissions from private-car commuting slowly increase.

In conclusion, the regulation of regional traffic volume can effectively reduce the cost of private vehicle commuting and reduce motor vehicle emissions. Vehicle flow rate in roadway q has no significant impact on the school-bus or the joint-commuting model.

#### 5.3.3. Sensitivity of Parking Spaces

**Figure 15:**
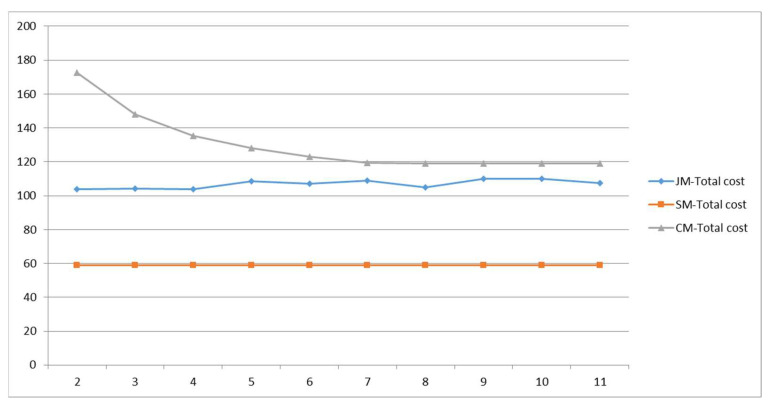
Total cost variations with parameter s.

The number of parking spaces affects the collection and distribution efficiency and time; we will explore the impact of local parking organization optimization on the model. The result is shown in Figure 15; with the increase of parking spaces at the entrance of the school, the cost of private-car commuting decreases and reaches a stable state when s = 7, but the joint-commuting cost is still lower than the private-car commuting cost. Due to the randomness of the algorithm, the cost of the joint-commuting model fluctuates in a small range and is basically not affected by the variation of the parking space, which demonstrates the stability of the model. The school-bus commuting costs remain the same because the size of the student scale remains the same.

**Figure 16:**
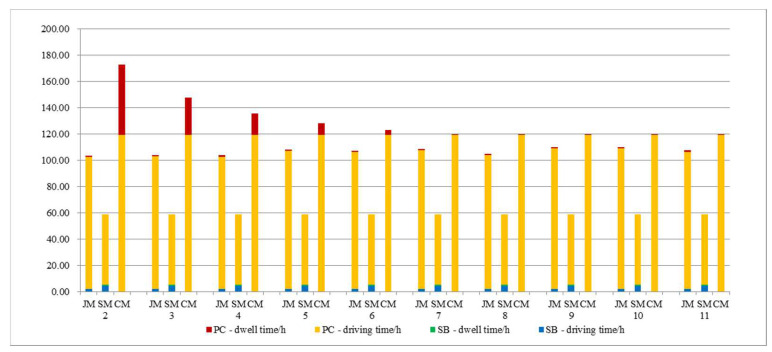
Various cost variations with parameter s.

It can be seen from Figure 16 that the dwell time of private-car commuting decreases with the increase of school parking spaces; when s > 6, it is less than the joint-commuting dwell time; the number of parking spaces can have a significant impact on commuting model choices.

**Figure 17:**
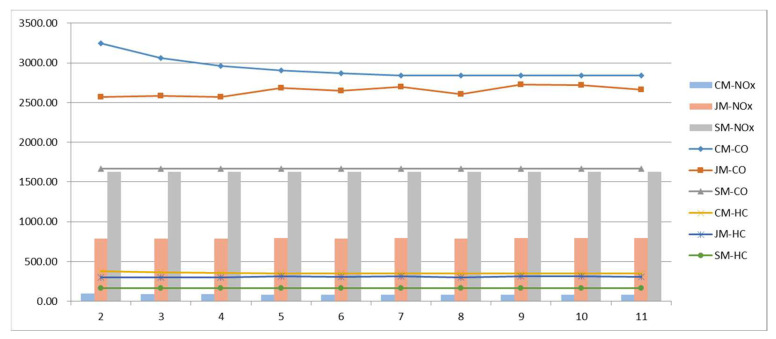
Pollutant emissions variations with parameter s.

Figure 17 shows vehicle emissions data for three commuting models. The data show two types of CO and HC emissions ranked from high to low: private-car commuting, joint commuting, and school-bus commuting. The ranking of NO_x_ emissions from high to low is school-bus commuting, joint commuting, and private-car commuting. As parking spaces increase, emissions from private-car commuting slowly increase.

To sum up, by reasonably increasing the temporary parking spaces in front of the school entrance, private-car commuting costs and the emissions from motor vehicles can be effectively reduced. The number of parking spaces has no significant impact on the school-bus and joint-commuting models, but it has a greater impact on the choice of commuting mode.

#### 5.3.4. Sensitivity of Drop-Off Time

**Figure 18:**
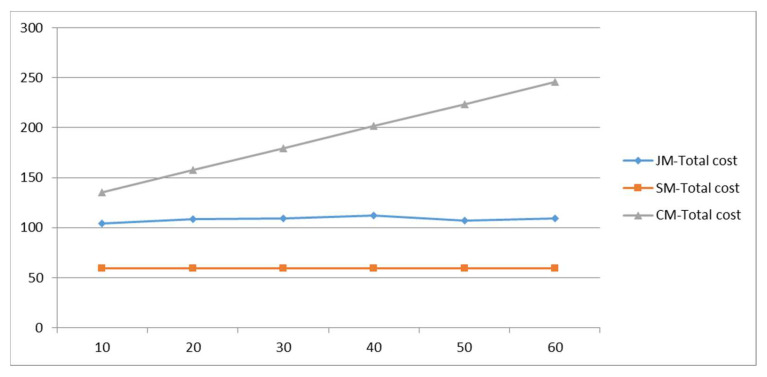
Total cost variations with parameter $t_{u}$.

The drop-off time reflects the organizational efficiency of vehicle parking in front of the school; the total cost of the three commuting models varies with the drop-off time as shown in Figure 18. The results show that, with the increase in the drop-off time, the cost of private-car commuting shows a linear growth trend, and the growth rate is fast. Due to the randomness of the algorithm, the cost of the joint-commuting model fluctuates in a small range and is basically not affected by the variation of the drop-off time, which demonstrates the stability of the model. The school-bus commuting costs remain the same because the size of the student scale remains the same.

**Figure 19:**
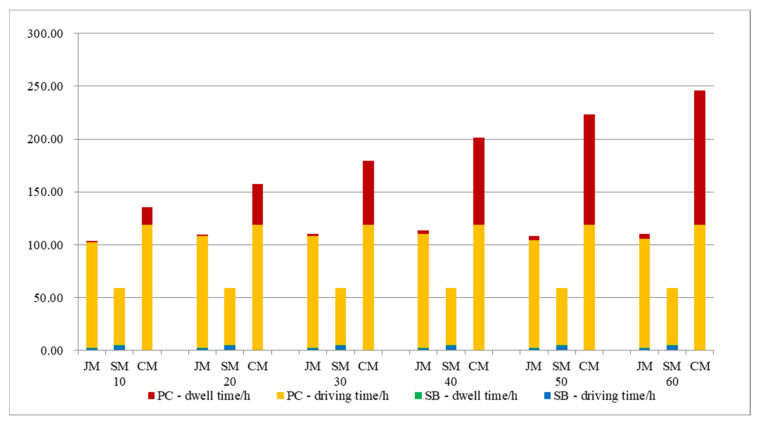
Various cost variations with parameter $t_{u}$.

It can be seen from Figure 19 that, with the increase in the drop-off time, the dwell time of private vehicle commuting increases significantly, indicating that the drop-off time is one of the important reasons for traffic congestion at the school entrance, while the dwell time of joint commuting shows a slowly increasing trend, which does not have an obvious impact.

**Figure 20:**
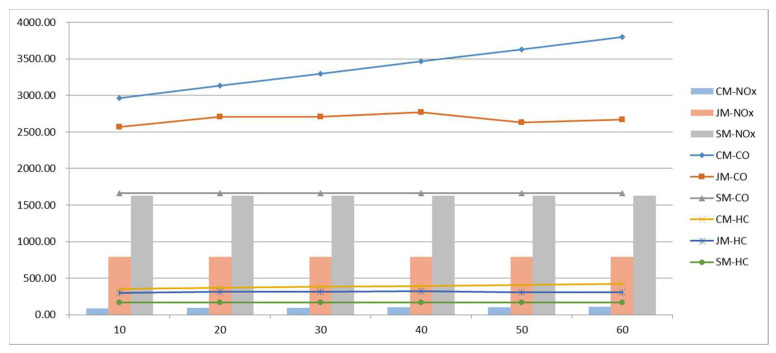
Pollutant emissions variations with parameter $t_{u}$.

Figure 20 shows vehicle emissions data for the three commuting models. The data show that two types of CO and HC emissions rank from high to low: private-car commuting, joint commuting, and school-bus commuting. The ranking of NO_x_ emissions from high to low is school-bus commuting, joint commuting, and private-car commuting. Emissions from private-car commuting tend to increase with the increase in drop-off time.

In conclusion, by effectively organizing the parking management at the school entrance and improving the utilization efficiency, private-car commuting costs and the emissions of motor vehicles can be reduced. The drop-off time has no significant effect on the school-bus or the joint-commuting model.

## 6. Conclusions

Based on the idea of “public–private cooperation” and “parking sharing”, this paper puts forward a two-phase, joint-commuting model that utilizes both school buses and private cars, and which aims to alleviate the traffic congestion around schools caused by private-car commuting on the basis of ensuring a certain service level. The proposed model takes into account parents’ costs and school-bus costs. The feasibility of the model is verified by numerical examples and sensitivity analysis. The results show that:

(1) Compared with the private-car commuting model, the joint-commuting model reduces the travel cost, especially the dwell time cost; the total cost is reduced by 23.33%, and the private-car cost is reduced by 25.15%, of which the dwell time during the school commuting phase is reduced by 92.29%. Joint commuting not only reduces the dwell time of parents, but it also reduces detours, which reduces the driving time in the school commuting phase by 23.36% and the driving time in the work or home stage by 7.44%.
(2) The results of sensitivity analysis show that the joint-commuting model has strong stability and is minorly affected by the traffic environment around the school and the arrival of families. School-bus commuting also has strong stability, and the total cost is lower than that of both joint commuting and private-car commuting.
(3) For the private-car commuting model, adopting staggered commutes to and from school can reduce the arrival rate of students. Regional traffic control strategies, similar to the morning rush-hour vehicle restrictions, and other measures to reduce the volume of traffic around schools, can reduce the dwell time of pickup vehicles. By setting up special parking spaces at the entrance of schools and organizing parking reasonably, the collection and distribution efficiency can be improved, and the traffic congestion around schools can be effectively alleviated.
(4) Compared with private-car commuting model, joint-commuting model can also effectively reduce motor vehicle pollutant emissions and reduce the impact of the system on the external environment. However, the results of the analysis show that the school-bus commuting model is still the most economical and environmentally friendly way to commute to school. For areas where private-car commuting accounts for a large proportion of travel, staggered commuting to school, traffic flow regulation, increasing parking capacity, and improving parking efficiency can be adopted to reduce system costs and impact on the environment during the commute period.

This paper is a preliminary exploration of the joint model. There are many limitations to this paper: It did not take into account the parents’ acceptance of the new model proposed in this paper and their preferences in choosing transfer stations; it did not study the traffic congestion at connecting stations, and it ignored the traffic conditions of different roads. More cost factors will be considered in future work, such as the travel costs of students, the maintenance costs of vehicles, the penalty costs caused by transfers, and the security costs. A survey will be carried out to grasp the preference of the parents and their level of acceptance of the new model. Otherwise, future research will involve the differences between leaving school and the time-varying characteristics of road traffic conditions. Finally, another future study will verify that the model is applicable to multiple school situations.

## Figures and Tables

**Table 1:** The parameters and decision variables in the mathematical model.

**Parameter**	**Parameter Meaning**
H	Set of home site
$P^{+}$	Set of transfer station
$P^{-}$	Set of school
P	Set of transfer stations and school
D	Set of workplace
M	Set of school bus
A	Set of family
Q	Capacity of school bus
$N_{j}$	Number of students at site *j*
$t_{jj^{\prime }}^{b}$	Time length of school bus from *i* to *j*
$n_{mj}$	Number of students picked up by school bus *m* at station *j*
$l_{mj}$	Number of students on school bus *m*
$Z_{jm}$	The time when school bus *m* arrives at station *j*
$T_{mj}^{f}$	Service time of school bus *m* at bus stop *j*
$t_{aij}^{c}$	The time it takes for family *a* to travel from *i* to *j*
$T_{aj}^{c}$	Dwell time of family *a* at transfer station *j*
$X_{aijk}$	If family *a* goes from *i* to transfer station *j* and then goes to *k*, $X_{aijk}$ = 1, otherwise 0
$X_{aiji}$	If family *a* goes from *i* to transfer station *j* and then goes home, $X_{aiji}$ = 1, otherwise 0
$y_{jj^{\prime }m}$	If school bus *m* goes from *j* to $j^{\prime }$,$y_{jj^{\prime }m}$ = 1, otherwise 0
**Decision Variable**	**Meaning**
$x_{aij}$	If family *a* goes from *i* to *j*, $x_{aij}$ = 1, otherwise 0

**Table 2:** Related information.

Parameter	Unit	Numerical Value
$t_{u}$	S	10
q	$\mathrm{veh}\cdot s^{-1}$	0.31, 0.54, 0.29, 0.29, 0.45, 0.36, 0.56, 0.32, 0.47, 0.44, 0.36, 0.49, 0.51, 0.41, 0.5, 0.46, 0.4, 0.43, 0.41
*S*	parking spaces	4, 25, 13, 21, 25, 22, 30, 19, 19, 11, 13, 20, 30, 14, 12, 19, 16, 20, 11
$v^{b}$	km/h	20
$v^{c}$	km/h	24
Δt	S	300
τ	S	3.75
h	S	2.65
Q	Seats	52
$T_{\max}$	S	1800

**Table 3:** The number of students at the transfer station.

Station Number	Number of Students (per)	Station Number	Number of Students (per)
0	6	10	7
1	5	11	14
2	18	12	18
3	19	13	11
4	17	14	12
25	18	15	14
6	18	16	12
7	13	17	14
8	14	18	9
9	13		

**Table 4:** School-bus route.

School Bus	Students per Bus	Route	Route Length (km)	Travel Time (min)
1	52	13–18–17–14–15–0	7.19	21.6
2	52	11–12–13–16–0	8.47	25.4
3	47	10–9–8–7–0	9.50	28.5
4	52	1–2–6–4–0	9.68	29.1
5	43	5–3–4–0	8.91	26.7

**Table 5:** Travel cost analysis of various modes in the joint-commuting model (h).

Analysis Index			Private-Car Commuting	School-Bus Commuting	Joint Commuting
School bus	Driving time		0	4.85	2.19
	Dwell time		0	0.63	0.28
	Subtotal		0	5.48	2.47
Private car	School commuting stage	Driving time	64.05	0	49.09
		Dwell time	16.26	0	1.25
	Work/home stage	Driving time	55.17	53.59	51.06
	Subtotal		135.48	53.59	101.40
Total			135.48	59.07	103.87

**Table 6:** Cost saving (%).

Analysis Index			Cost Savings of Joint Commuting Compared with Private-Car Commuting	Cost Savings of Joint-Commuting Compared with School-Bus Commuting
School bus	Driving time		/	54.93
	Dwell time		/	54.72
	Subtotal		/	54.88
Private car	School commuting stage	Driving time	23.36	/
		Dwell time	92.29	/
	Work/home stage	Driving time	7.44	4.74
	Subtotal		25.15	−89.21
Total			23.33	−84.31

**Table 7:** Emission coefficient in driving state.

Parameter	Vehicle Type	CO	HC	NO_x_
Comprehensive benchmark emission coefficient BEF	Private car	0.46	0.056	0.017
	School bus	1.62	0.054	8.64
Environmental correction factor φ	Private car	1.36	1.47	1.15
	School bus	1	1	1.06
Road traffic condition correction factor γ	Private car	1.26	1.25	1.13
	School bus	1.29	1.38	1.39
Deterioration correction factor δ	Private car	1.26	1.18	1.33
	School bus	1.43	1.48	1.25
Emission coefficient in driving state EF (g/km)	Private car	0.99	0.12	0.03
	School bus	2.99	0.11	15.91

**Table 8:** Emission coefficient in dwell state.

Parameter	Vehicle Type	CO	HC	NO_x_
Emission coefficient in dwell state (mg/s)	Private car	2.10	0.16	0.05
	School bus	42.73	0.25	20.66

## Data Availability

Not applicable.
